# Heritage language development in Spanish–English-speaking preschoolers: Influences on growth and challenges in the first year of English-only instruction

**DOI:** 10.1017/S030500092400045X

**Published:** 2024-09-27

**Authors:** Simona Montanari, Gabriela Simon-Cereijido, Jieru Bai, Kaveri Subrahmanyam

**Affiliations:** 1 California State University, Los Angeles; 2 University of North Florida

**Keywords:** heritage language development, dual language learners, Spanish, gender, maternal acculturation and enculturation

## Abstract

This study investigates the changes in the Spanish lexical and grammatical skills of 26 Spanish–English dual language learners during their first year of preschool. We also explore the impact of age, gender, and maternal cultural orientation on children’s language outcomes over time. The results show that, despite one year of English-only instruction, the children’s Spanish productions became more intelligible, lexically diverse, and grammatical between 3;7 and 4;7. However, Spanish productions were mostly limited to sentence fragments and contained errors in grammatical gender, verb morphology, object clitic pronouns, and prepositions. Girls had an advantage over boys, as attested by the higher lexical diversity, mean length of utterance, and grammaticality of their Spanish productions. Both maternal enculturation and acculturation predicted the grammaticality of children’s utterances, suggesting that mothers with high levels of orientation to both Latinx and American culture may be the most successful at promoting Spanish in the United States.

## Introduction

Immigrant populations across the world are often affected by language shift, a pattern of language use in which the prominence and use of the immigrant community’s native language decrease, leading to an increase in the prominence and use of the societal language (Silva-Corvalán, 1991). Typically, language shift occurs over time and across generations, with the first generation of members from the immigrant group being fluent in their native language but limited in the host country’s language, the second generation being bilingual but dominant in the societal language, and the third generation speaking only the host country’s language. While language shift is gradual and occurs across generations, language use and proficiency changes can also occur within individual speakers and one’s lifespan, in a process called language attrition. Language attrition involves the gradual loss of previously acquired language abilities (Schmid & Köpke, 2009). In the case of young children exposed to both a majority and a minority language, the development of the minority language may stall, resulting in incomplete acquisition compared to monolingual speakers of the target language (Montrul, 2018). Language attrition and incomplete acquisition tend to occur in socio-political contexts characterized by a “minority-majority language dichotomy,” where differing values are attributed, either explicitly or implicitly, to each of these languages (Anderson, 2022, p. 196). This is clearly the case in the United States (US), where the higher-status societal language, English, is not only ever-present in all domains (on the screen, in the street, at school, etc.) but also necessary for professional and educational advancement.

Understanding language attrition and incomplete acquisition in contexts where multilingual speakers constitute a large segment of the population is important for educators and policymakers. For instance, in the US – especially its urban areas – many individuals use more than one language daily, and Spanish alone is spoken by over 40 million speakers (www.census.gov). In California, Spanish-speaking Latinx represent nearly one-third of the entire population, and 40% of Los Angeles County residents speak Spanish. In these families, Spanish may be the primary home language; yet children hear both Spanish and English from early on and are formally and consistently exposed to English in preschool or kindergarten, while their Spanish is still being developed. The number of such children in US schools – dual language learners (DLL, Paradis et al., 2021) – has doubled in recent years, and Spanish-speaking children represented 75.5% of all English learners in 2020 (National Center for Education Statistics, 2023). These statistics suggest that Spanish–English DLLs make up a large proportion of students in the classroom, and they may be at risk of attrition or incomplete acquisition of the first (L1) or heritage language (HL) as they are educated in the societal language.

Since it has been argued that second language (L2) acquisition is mediated by L1 learning (see Paradis et al., 2021, for a summary), understanding Spanish development among Spanish–English DLLs has significant implications for English language development. Indeed, it has been proposed that as children acquire knowledge and skills in their HL, they also abstract language-independent information that they can apply across learning contexts, a process that may facilitate L2 acquisition (structural sensitivity theory, Kuo & Anderson, 2012; Kuo et al., 2016). This is because, while learning one language, a child acquires a set of skills and knowledge, including conceptual knowledge and syntactic structure, that will support the acquisition of the same concepts and structures in L2. Several studies of young DLLs in different parts of the world have found, indeed, that children who have stronger oral language abilities in their L1 at the beginning of preschool display stronger oral language abilities in their L2 by the end of preschool (Castilla et al., 2009; Grøver et al., 2018; Jackson et al., 2014; Winsler et al., 2014). Pace et al. (2021) further found, in a heterogeneous group of Spanish–English bilingual preschoolers, that grammar comprehension and word learning abilities in Spanish were related to grammatical and word learning processes in English. The authors concluded that proficiency in Spanish can particularly provide a critical springboard for the acquisition of English in this population, since the two languages share structural features that may be mutually informative for word learning (e.g., SVO syntax in this case). Furthermore, L1 proficiency has been shown to facilitate communication with family and community members, with competence in this language playing an important role in DLLs’ socio-emotional development (Winsler et al., 2014), emerging cultural identity, and formation of a positive self-concept (Oh & Fuligni, 2010; see also De Houwer, 2020, for a review). Therefore, given the interdependence of L1 and L2 development and the positive contribution of HL competence to identity development, continued growth and proficiency in L1 Spanish is inherently important to DLLs in the US.

Yet, studies of language attrition among Spanish–English DLLs in the US are scarce and dated. In a review on the topic, Anderson (2022) argues that the changes in language exposure patterns that children experience during the preschool years, when they transition from being cared for at home in Spanish to being educated in preschool in English, are the major culprits of L1 attrition. For instance, changes in the relative use of each language, with a transition towards greater use of English across domains, limit instances in which the child hears and uses Spanish. In turn, limited Spanish input and output prevent further development and maintenance of acquired L1 language skills, especially in the lexicon and grammatical system. At the same time, as children are educated in English, they become aware that this is the language of prestige and power and the preferred language in the community, while Spanish is a minority language spoken only at home and by a segment of speakers in their community, a language “disfavored in typical American classrooms” and “within a sociopolitical context that does not support maintenance of the home language” (Gutiérrez-Clellen et al., 2009, p. 105). This situation slows down HL development and accelerates attrition and shift to the societal language and culture. Changes in language exposure and use patterns further interact with many demographic, social, and individual variables, resulting in differing degrees of language attrition and maintenance.

It is the goal of this study to examine the changes in the Spanish lexical and grammatical skills of 26 Spanish–English DLLs from Los Angeles, California, after formal exposure to English began in preschool at around 3;7 years of age. In particular, we investigate whether the children’s Spanish vocabulary and grammatical system shows growth or attrition from the beginning of their first year in preschool to a year later when the children are raised and schooled in a sociopolitical context that significantly favors the majority language, English, at the expense of the children’s HL, Spanish (Anderson, 2022). Since the process of language attrition is affected by various external and internal factors, we also investigate the extent to which other variables affect this process, including children’s gender and maternal cultural orientation.

### Variables associated with language attrition

Family language policy (including parental and familial language use) and attitudes toward the use of L1 and L2 are critical factors that foster or hamper HL development among DLLs (see Quay & Montanari, 2016, for a review). Families that primarily use the HL increase children’s opportunities for HL development and indirectly positively influence their perception of it. On the other hand, families who primarily use the societal language limit children’s access to the HL, adversely affecting its development and, possibly, children’s attitudes towards it. In fact, research has shown that L1 attrition occurs more rapidly when parents are bilingual – and thus speak the societal language – because, in this context, not enough communication in the HL may be provided for children to become active bilinguals and children can use the L2 and still be understood by parents (Quay & Chevalier, 2019).

Children’s early immersion into English-only preschool programs may also accelerate HL attrition. Wong-Fillmore (1991) surveyed over 1,100 immigrant families and found that children who attended English-only preschool programs were much more likely than children enrolled in bilingual programs to experience L1 attrition. Indeed, HL competence before children enter school is no guarantee of later bilingualism when schooling occurs early and exclusively in the majority language. On the contrary, since the vast majority of DLLs in the US attend schools in which English is the primary or sole language of instruction, an accelerated growth in English concomitant to a deceleration in the development of Spanish after entering the school system are the norm in this population (Hammer et al., 2012; Hoff et al., 2018; Montanari et al., 2019, 2022; Rojas & Iglesias, 2013). This is because, as children enter school, contexts for speaking English increase, whereas those for using Spanish diminish.

Other demographic, social, and individual variables interact with parental language use and attitudes and early English immersion to affect the process of L1 maintenance in DLLs. For instance, several studies have found that gender predicts successful HL acquisition (Arriagada, 2005; Portes & Hao, 1998; Portes & Rumbaut, 2001; Rojas & Iglesias, 2013; Zentella, 1997). Zentella (1997), in an ethnographic study of Puerto Ricans in New York, found that females tended to have higher levels of Spanish maintenance and proficiency than males from the same neighborhood, and Spanish language use was associated more with female than male domains. Portes and Hao (1998) also documented better performance on HL proficiency measures among second-generation females than males, a finding that was interpreted as resulting from girls spending more time with their parents and thus receiving more exposure to the HL. Rojas and Iglesias (2013) further studied gender differences in HL development in a longitudinal study of 1,723 Spanish–English-speaking children aged 5;7 years at the beginning of their first three years of formal schooling. Boys and girls showed similar growth patterns in Spanish and English, although the shapes of their trajectories differed. In Spanish, girls had higher initial proficiency levels than boys, and their growth rates were consistently higher across different language measures throughout the study. In English, boys and girls started at similar levels, but girls showed faster growth in the spring semesters. Hammer et al. (2009) did not find that gender predicted vocabulary and early literacy skills in either Spanish or English among Spanish-speaking preschoolers and kindergarteners. However, the study documented differences in language usage based on the gender of the child: mothers of daughters were eight times more likely to speak to them using more or all Spanish when compared to mothers of sons. Mothers of sons were twice as likely to speak to their sons using more or all English than the mothers of girls. Hammer et al. (2009) speculated that these language use patterns may derive from traditional gender norms where girls, but not boys, are socialized into staying at home and becoming parents (hence being immersed in the home Spanish environment) (Zentella, 1997).

Research on Latinx families also suggests that parental levels of acculturation (i.e., orientation to American culture) and enculturation (i.e., orientation to Latinx culture) (Gonzales et al., 2004) relate to L1 use in the home and children’s HL learning. Typically, English acquisition and usage are evidence that an immigrant parent has acculturated to mainstream society, and their children may be more exposed to English than the HL. In contrast, the retention and more frequent use of the L1 by the immigrant parent may indicate lower levels of involvement and acculturation to the host country but increased HL learning in children (Phinney & Flores, 2002). Boyce et al. (2013) found a positive and significant correlation between maternal acculturation and total vocabulary size (including both English and Spanish words) in Spanish–English bilingual toddlers at 24 and 36 months of age. However, the study did not separately examine the influence of maternal acculturation and enculturation on children’s English and Spanish language outcomes, respectively. Cote and Bornstein (2014) studied bilingual American children, aged 20 months, born to Korean, Japanese, and South American immigrant mothers. They found that more acculturated mothers exposed their children more to English, leading to larger English vocabularies in the children. On the other hand, more enculturated immigrant mothers reported increased use of the HL with their children, resulting in larger HL vocabularies for the children.

### Lexical and grammatical patterns of language attrition in Spanish–English dual language learners

Few studies have specifically looked at L1 attrition in young Spanish–English-speaking children. Early investigations were case studies of a few children (Anderson, 1999a, 1999b, 2001); other seminal studies focused on adults. For example, Montrul (2008, 2016) documented extensive and often subtle differences in the lexical and morphosyntactic competence of adult Spanish HL speakers with respect to monolingual Spanish speakers. In the author’s words (Montrul, 2012, p. 4), by the time HL learners become adults, they display “non-native like competence and use of the language, better ability with receptive than productive language, non-uniform levels of proficiency, and linguistic gaps that resemble the patterns attested in second language acquisition (in gender agreement, verb paradigms, pronouns, case marking, word order, prepositions, etc.).” As a matter of fact, Montrul (2016) points out that many of the differences between HL speakers and monolinguals may be due to differential acquisition of the L1 because these speakers may not have had the opportunity to fully acquire the morphosyntactic aspects of their L1. See Silva-Corvalán (2014, 2018) for a similar argument.

Other studies that can inform us about L1 attrition have focused on bilingual development in DLLs, examining overall measures of language competence (often measured through parental reports) rather than specific lexical and syntactic patterns. For example, Hoff et al. (2018), who studied the English and Spanish lexical and grammatical abilities of Spanish–English bilingual children between 30 and 48 months using parental reports, documented a negative relationship between level of English skills (either in vocabulary or grammar) and subsequent Spanish growth, suggesting that children with higher English proficiency early on were more likely to display lower Spanish competence with time. These results were interpreted as evidence that growing competence in the societal language threatens children’s continued acquisition of the HL. Hammer et al. (2008, 2012) also investigated the Spanish and English developmental trajectories of bilingual preschoolers from preschool entry at around 3;6 years of age to program exit at approximately age 5. Hammer et al. (2008) focused on receptive vocabulary and language comprehension abilities and found that children’s raw scores on the English receptive vocabulary test accelerated, while children’s standard scores on the Spanish language comprehension measure decelerated after an initial period of linear growth. Thus, despite Spanish exposure at home, the participants appeared to be losing their Spanish comprehension abilities after a significant change in the language environment occurred. Hammer et al. (2012) found that simultaneous bilinguals began preschool with comparable productive vocabularies in the two languages but English vocabularies were larger than Spanish vocabularies by the end of preschool, and growth in English was strong, whereas it was weak in Spanish. On the other hand, sequential bilinguals, who predominantly began to acquire English in the preschool program, had larger Spanish vocabularies throughout preschool. However, there was stronger growth in English than in Spanish, suggesting, as in other studies, accelerated growth in English concomitant to a deceleration in the development of Spanish after entering preschool (Hammer et al., 2012; Hoff et al., 2018; Montanari et al., 2019, 2022). It is important to point out, however, that for children who spoke predominantly Spanish at home, attending English-language preschool did not result in a dominant language shift and/or L1 attrition.

Research on how L1 attrition affects production abilities in developing Spanish–English DLLs is scarce and mostly limited to case studies. These studies have shown that the lexicon and the grammatical system are the most affected areas of development during this process (Anderson, 1999a, 1999b, 2001; Castilla-Earls et al., 2019; Guiberson et al., 2015; Hiebert & Rojas, 2021; Silva-Corvalán, 2014, 2018). Indeed, the reduction of L1 input and use lowers the speed and accuracy of lexical retrieval, eventually causing loss of lexical knowledge and a narrowing of the vocabulary (see Anderson, 2022, for a review). Early patterns of lexical loss include a reduction in the production of nouns, followed by a reduction in the production of verb lexemes (Anderson, 2022). For instance, in a longitudinal case study of a Spanish–English bilingual child who was experiencing L1 attrition, Anderson (1999a) documented a significant decline in the use of different nouns and action verbs over time and showed an increase in the use of general terms such as the demonstrative pronouns *éste*, “this,” or *ése*, “that” (e.g., *quiero ése*, “I want that,” instead of *quiero el juguete*, “I want the toy”). Other studies have documented an increase in code-switching to English to make up for Spanish vocabulary gaps (Anderson, 1999a, 1999b, 2001; Guiberson et al., 2015; Hiebert & Rojas, 2021; Montanari et al., 2019). Relatedly, Hiebert and Rojas (2021), who examined the trajectories of Spanish language growth and loss in 34 Spanish–English DLLs from preschool to kindergarten, found that lexical diversity in Spanish (as measured by the Moving-Average Type-Token Ratio, MATTR) suffered from a significant decline over time, especially when code-switched utterances were excluded from the analyses.

Reduction in input and output also has an impact on the grammatical skills of Spanish–English DLLs, resulting, in particular, in a progressive reduction of productivity and inflectional morphology with possible regularization of irregular forms, errors at the morphosyntax-semantics-pragmatics interface, including with determiners and subject pronouns, and the transfer of L2 syntactic structure to the L1 (Anderson, 1999a, 1999b, 2001; Silva-Corvalán, 2014, 2018; see also Montrul, 2018, for a review). Hiebert and Rojas (2021) specifically documented a significant deceleration of mean length of utterance in words (MLUw) in Spanish coupled with a decrease in the proportion of grammatical utterances (PGU) between preschool and kindergarten. Similarly, Castilla-Earls et al. (2019) reported a decrease in PGU in Spanish between kindergarten and second grade even in children receiving Spanish–English bilingual instruction. It has been reported that features of the noun phrase and verb morphology are particularly affected in HL development when children experience a shift from Spanish to English dominance (Castilla-Earls, Pérez-Leroux, et al., 2020; Montrul, 2022).

At the noun phrase level, mismatches in gender agreement and case are frequent (Anderson, 1999a, 1999b; Anderson & Márquez, 2009; Hiebert & Rojas, 2021). Indeed, studies of heritage Spanish-speaking children in the US experiencing L1 attrition reveal that the main mismatches are in the use of a masculine article with a feminine noun or vice versa (e.g., *el mesa, “the (masc.) table (fem.),” rather than *la mesa*, “the (fem.) table (fem.),” or *la perro, “the (fem.) dog (masc.),” rather than *el perro*, “the (masc.) dog (masc.)”) or the use of a singular article for a plural one (*un perros, “a dogs”) (Castilla-Earls, Pérez-Leroux, et al., 2020; Martinez-Nieto & Restrepo, 2023). At the verbal phrase level, changes affect the use of tense, aspect, mood, person, and number distinctions. Tense errors occur when a required tense (e.g., past tense as in *se fue*, “he left”) is replaced by another tense, for example, the simpler and more default present tense (*se va*, “he leaves”). Examples of aspect errors include the use of a perfective tense (such as the perfect tense in *comí*, “I ate”) when an imperfective tense is required (i.e., the imperfect tense in *comía*, “I used to eat”). Finally, examples of mood errors include the use of the indicative mood (e.g., *no quiero que lo* *sabe, “I don’t want that s/he knows”) for the subjunctive mood (e.g., *no quiero que lo sepa*, “I don’t want that he/she knows”) in instances in which the subjunctive form is the necessary one (Anderson, 2001; Silva-Corvalán, 2014, 2018; see also Montrul, 2018, for a review). Person and number distinctions are also aspects of grammar that are adversely affected by L1 attrition after early L2 exposure, resulting in errors with subject-verb agreement. For example, Anderson (2001) reported that singular forms replaced plural ones (e.g., *duerme*, “he/she sleeps” for *duermen*, “they sleep”), and the third person singular form was overextended to all other forms (*quiere*, “he/she wants,” for *quieres*, “you want”). Thus, the trend was a reduction of the person/number paradigm and the collapsing of all forms to a general, single one: the more default third person singular form. The regularization of irregular verb forms (i.e., *sabo* for *sé*, “I know”) has also been documented in the extant literature, although children’s patterns of overregularization have been shown to be inconsistent and were evidenced with some, but not all, irregular verb forms (Anderson, 2001; Silva-Corvalán, 2014, 2018), suggesting that factors such as complexity, saliency, and frequency of occurrence in the input and output may be responsible for this pattern.

### The present study

The goal of this study is to examine the changes in the Spanish lexical and grammatical productive skills of 26 young Spanish–English DLLs from the beginning of their first year of preschool (average age 3;7) to a year later. Since previous studies on bilingual development in DLLs have focused on children’s language abilities as reported by parents (Hoff et al., 2018), on receptive vocabulary and language comprehension skills (Hammer et al., 2008, 2012), or on a limited number of production variables in preschoolers (Hiebert & Rojas, 2021) and school-aged children (Castilla-Earls et al., 2019), we examine the patterns of change in HL proficiency during the preschool years as measured by a wide range of lexical and grammatical measures in Spanish spontaneous speech, including 1) the number of complete and intelligible utterances (NCIU), 2) the total number of words (TNW), 3) the number of different words (NDW), 4) the mean length of utterance in words (MLUw), 5) the proportion of utterances with verbs (PUV), 6) the number of omitted words (NOW), and 7) the proportion of grammatical utterances (PGU).

Furthermore, since the process of language development and attrition is affected by a variety of external and internal factors that have been scarcely investigated (e.g., maternal cultural orientation) or poorly understood (e.g., gender), we run multiple linear models to investigate the extent to which demographic, social, and individual variables – in particular, age, gender, and mother’s enculturation and acculturation – predict each of the outcome proficiency variables. Finally, because the areas of language production that are most affected by L1 attrition are the grammatical system (Anderson, 2022), we conduct a qualitative analysis of children’s deviations from standard Spanish grammatical production between ages 3;7 and 4;7. We thus ask the following research questions:

1. To what extent do children’s Spanish lexical (TNW, NDW) and grammatical (NCIU, MLUw, PUV, NOW, PGU) skills displayed in spontaneous speech change during the first year of English-only instruction between ages 3;7 and 4;7?
2. Do children’s age, gender, and mothers’ enculturation and acculturation predict each of the outcome proficiency variables?
3. To what extent do children’s production of articles, copula verbs, and prepositions at ages 3;7 and 4;7 deviate from standard Spanish grammatical forms?

Based on the extant literature (Castilla-Earls et al., 2019; Hammer et al., 2012; Hiebert & Rojas, 2021; Hoff et al., 2018; Montanari et al., 2019, 2022), we hypothesize that the children’s Spanish lexical and grammatical skills may show limited growth between ages 3;7 and 4;7, as children are introduced to formal English-only schooling. We also expect that features of the noun phrase and verb morphology will be particularly affected by increased English exposure and a reduction of Spanish input. We are unable to put forward a conclusive hypothesis as to the extent to which children’s age, gender, and mothers’ cultural orientation will predict children’s Spanish productive skills given that studies have produced mixed results as to the relevance of these factors.

## Method

### Participants

This study used data from a larger longitudinal investigation of dual language development among Spanish–English-speaking preschoolers attending Head Start programs in Southern California. We focused on 26 children (11 males, 15 females, the full sample included more females) for whom detailed child and maternal information was available in addition to Spanish spontaneous speech samples collected at preschool entry, when the children were on average 3;7 of age (age range: 3;1-4;1), and a year later, when they were on average 4;7 (age range: 4;1-5;1). All children were developing typically and had no hearing, speech, language, cognitive, or neurological deficits based on parental reports and screening tests administered by the programs. All children were born in the US but came from Spanish-speaking families with parents born in Mexico. A questionnaire administered to the mothers in Spanish at the beginning of the study was used to collect detailed demographic and language use information, including the language spoken by the mother to the child (i.e., language input), the language spoken by the child to the mother (i.e., language output) and the language spoken between siblings (both input and output). Table 1 reports this information. As can be seen, Spanish was reported to be the primary home language and the children’s native language, as well as the language primarily spoken between each mother and her child. However, all participants were also exposed to English through the media, the larger community, and, as shown in the table, their siblings. The children came from low socioeconomic (SES) backgrounds, as determined by their eligibility to attend the Head Start program, and the mothers had limited education with most of them having completed primary or secondary school. Most mothers were not employed at the time of the study and thus took care of their children.

#### Procedures

In order to measure mothers’ cultural orientation, mothers were administered the Acculturation Rating Scale for Mexican Americans-II (ARSMA-II, Cuéllar et al., 1995), which independently assessed their orientation to Mexican (enculturation) and Anglo-American culture (acculturation). The scale consists of an Anglo orientation subscale (AOS) with 13 items and a Mexican orientation subscale (MOS) with 17 items that assess various aspects of cultural orientation, including language use and preferences, ethnic identity and classification, cultural heritage and ethnic behaviors, and ethnic interaction. Each question is scored on a Likert scale from 1 (“not at all”) to 5 (“extremely often or almost always”). Mean scores for each subscale are calculated by adding the scores of all items and dividing it by the total number of questions. Higher AOS and MOS scores represent higher levels of acculturation and enculturation, respectively, whereas lower scores represent less orientation to that specific culture. Reliability and test-retest reliability for ARSMA-II scales are high as indicated by −0.83 and 0.88 correlations for the AOS and MOS, respectively (Cuéllar et al., 1995). Correlations between acculturation scores from the original ARSMA and those from the ARSMA-II have further revealed strong construct and concurrent validity as well as high convergent validity for the ARSMA-II, suggesting that it is a valid and reliable measure to assess acculturation among Mexican-Americans (Jimenez et al., 2010).

Table 1 shows the mothers’ enculturation and acculturation scores. The maternal mean enculturation score was 4.48 (*SD*: 0.37; range: 3.18-5.00), suggesting that mothers were “very Mexican oriented,” a categorization that accurately reflected their status as recent immigrants in a region characterized by a large Mexican community. In contrast, their mean acculturation score was 2.29 (*SD*: 0.79; range: 1.08-4.23), which indicated a more limited orientation to American culture. Thus, as shown by the standard deviations and ranges, mothers differed substantially in how much they were oriented to American culture, whereas they were more uniform in their Mexican orientation.

In order to examine children’s Spanish lexical and grammatical skills, we collected naturalistic speech samples at preschool entry and a year later during which children interacted with a research assistant who, although bilingual, posed as a monolingual Spanish speaker. Analysis of spontaneous language, which allows for a naturalistic observation of a child’s representative language skills, is one of the standard strategies for the assessment of child language (Gutiérrez-Clellen et al., 2000; Simon-Cereijido & Gutiérrez-Clellen, 2007). While research with school-age children has particularly relied on narrative samples (Rojas & Iglesias, 2013), language samples produced during play have long been used to document, in an effective and ecologically valid way, the expressive language development of preschoolers with and without language disorders (Binger et al., 2016).

Before collecting the data, the research assistant spent time in the Head Start classroom in order to familiarize herself with the children. During the data collection, each child was individually taken to a quiet room and recorded for approximately 45 minutes while interacting with the research assistant around a set of age-appropriate toys and books, including a food set, a car set, a doll set, a farm play set as well as the books *Frog Where Are You?* (Mayer, 1969) and *A Boy, a Dog, and a Frog* (Mayer, 1967). Because the goal was to elicit spontaneous speech, the research assistant, who was trained in eliciting complex language, interacted with the children naturally, asking open-ended questions that focused on the toys/books at hand but also on the child’s interests and leads. While Spanish and English speech samples were collected at both times, the current study is based only on the Spanish samples. See Montanari et al. (2018, 2019, 2021, 2022) for further details on the data collection protocol and the children’s speech and language skills in both Spanish and English.

#### Transcription and coding

The speech samples were transcribed orthographically by two research assistants and reassessed by a third transcriber until consensus on all transcriptions was reached. Then each transcription was coded and analyzed using Systematic Analysis of Language Transcripts (Miller & Iglesias, 2017). Since we were interested in examining the children’s grammatical abilities, we coded the first 100 multi-word utterances of each sample, excluding single words, for grammatical errors at the word and utterance levels. Traditionally, language samples with 50 to 100 utterances have been considered reliable for providing data that is representative of a child’s language skills (Miller et al., 2011; Paul & Norbury, 2012). As shown in the example (1) below, errors at the word level (EW) included errors with Spanish morphological production, omissions (marked with *), and overgeneralizations (EO), whereas errors at the utterance level (EU) mainly included word order errors.

1. Child: yo no sabo|saber[EO] qué es|ser[EW:son] esos.
  I don’t know[EO] what those is[EW:are].
  Child: (pero eso) pero voy|ir *a[EW] hacer otro *para[EW] mi papá.
  (but that) but I’m going *to[EW] make another *for[EW] my dad.
  Child: y es|ser un huevo donde hace|hacer [EU].
  And it’s an egg where it makes [EU]. (For “It’s where we make an egg”)

A second transcriber performed inter-rater reliability of coding, and disagreements were discussed by re-examining the transcriptions until consensus was reached. We then used SALT to automatically generate the lexical and grammatical proficiency measures that have been previously used to evaluate oral language samples and identify language attrition (Anderson, 1999a, 1999b, 2001; Castilla-Earls et al., 2019; Guiberson et al., 2015; Hiebert & Rojas, 2021), including: 1) the number of complete and intelligible utterances (NCIU), 2) the total number of words (TNW), 3) the number of different words (NDW), 4) the mean length of utterance in words (MLUw), 5) the proportion of utterances with verbs (PUV), 6) the number of omitted words (NOW), and 7) the proportion of grammatical utterances (PGU). We excluded abandoned utterances, unintelligible segments, and utterances with code-switching from these analyses as described in previous research and the SALT manual (Gutiérrez-Clellen et al., 2000; Hiebert & Rojas, 2021; Miller & Iglesias, 2017; Simon-Cereijido & Gutiérrez-Clellen, 2007).

#### Data analysis

Statistical comparisons of changes in children’s Spanish lexical and grammatical productive skills between 3;7 and 4;7 were completed using the Statistical Package for the Social Sciences (SPSS Statistics Version 27). Specifically, we ran mixed linear models (MLM) to investigate the extent to which 1) time of outcome measurement, 2) age at first measurement, 3) gender, 4) maternal enculturation, and 5) maternal acculturation predicted each of the outcome proficiency variables. MLM can compare change over time while allowing differences across different participants. The aforementioned five predictors were treated as the fixed effects, and time of measurement was treated as random effect. Because there were only two data points, only linear models were estimated for each outcome measurement.

We supplemented our quantitative analysis with a qualitative analysis of the non-standard grammatical productions of the children from their entry into preschool to one year later. We specifically focused on identifying and extracting utterances that contained grammatical errors. We recorded the frequency of ungrammatical usage of articles, object clitic pronouns, copula verbs, verbs (excluding copula verbs), and prepositions for each individual child. In the few cases in which we could not determine the intended target words based on the transcript, we excluded the nonstandard productions from the qualitative analysis.

To compare the children’s nonstandard productions at two different time points, we employed paired *t*-test analyses. Additionally, we conducted a frequency analysis to provide a more detailed characterization of the grammatical errors. This analysis involved coding instances of omissions and mismatches in gender and number for articles. For copula verbs and prepositions, we coded instances of omissions and substitutions.

## Results

**Figure 1.**
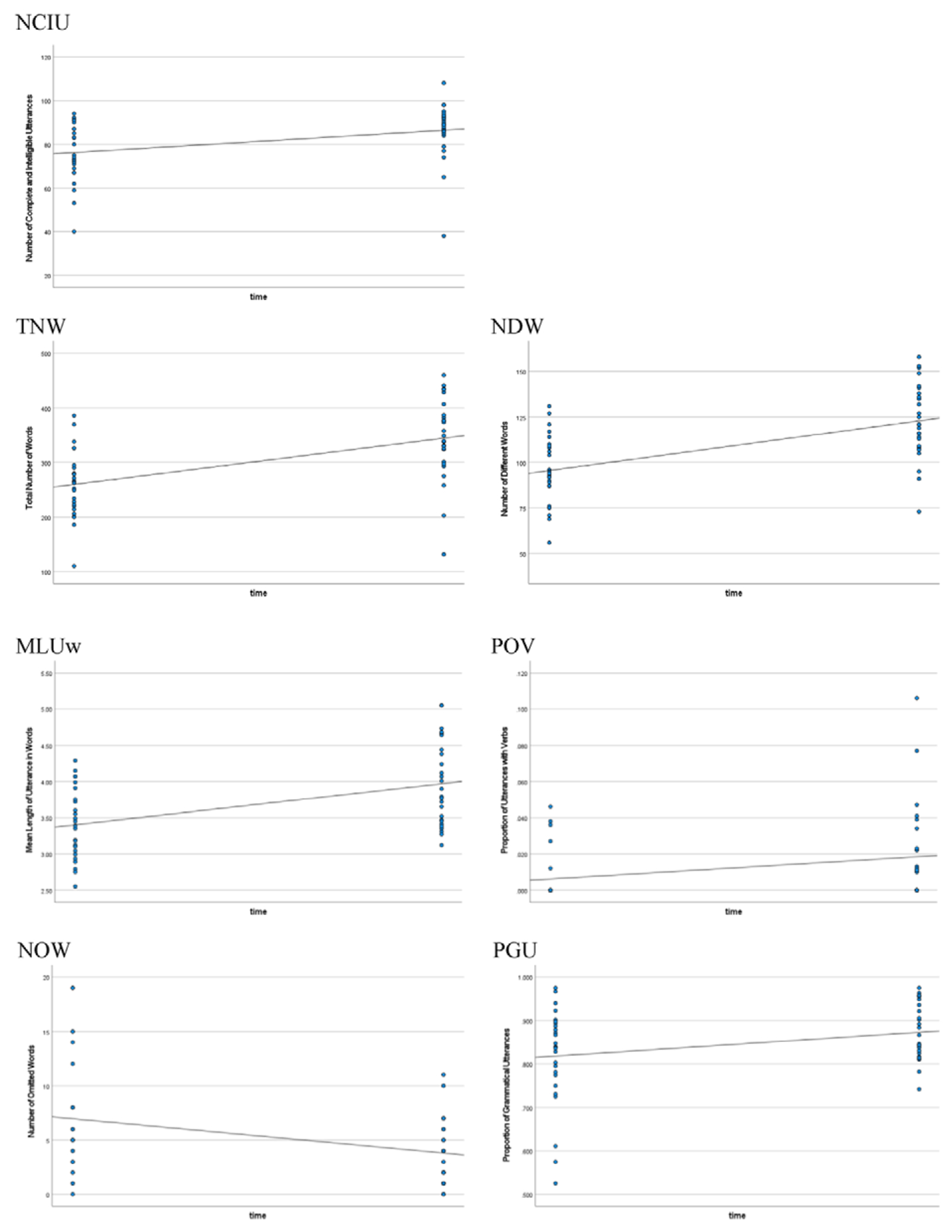
Individual Mixed Linear Model for Each Outcome Variable: 1) Number of Complete and Intelligible Utterances (NCIU), 2) Total Number of Words (TNW), 3) Number of Different Words (NDW), 4) Mean Length of Utterance in words (MLUw), 5) Proportion of Utterances with Verbs (PUV), 6) Number of Omitted Words (NOW), and 7) Proportion of Grammatical Utterances (PGU). *Note:* Number of complete and intelligible utterances (NCIU); Total number of words (TNW); Number of different words (NDW); Mean length of utterance in words (MLUw); Proportion of utterances with verbs (PUV); Number of omitted words (NOW); Proportion of grammatical utterances (PGU)

Multiple MLM models were run for each outcome variable with different fixed effects, random effects, covariance type, and with/without the interactions of effects. However, in order to be consistent across the seven outcome variables, we used the same procedure for all outcome variables. Schwartz’ Bayesian Criterion (BIC) was used to select the best model fit. Finally, we decided to include five independent variables as the fixed effects and did not allow the interactions among them: time, age, gender, mother’s enculturation (Mexican culture orientation), and mother’s acculturation (American culture orientation). Time was also treated as the random effect to allow for different growth pace for individual participants and control for possible dependence due to repeated measures. For covariance type, scale identity was used, which assumes no interactions between intercepts and slopes. This procedure resulted in better model fit for all outcome variables (see Figure 1). However, it may not be the best model fit for each individual outcome variable. Please refer to Table 2 for the outcome variables’ means and standard deviations at each timepoint and Table 3 for a summary of the regressions’ coefficients of the fixed effects for all the outcome variables. The supplemental materials contain the model summaries in full.

### Number of complete and intelligible utterances (NCIU)

The only significant fixed effect predictor of the number of complete and intelligible utterances was time (NCIU, Figure 1). The coefficient of time was 10.19, which indicates a predicted increase of 10.19 complete and intelligible utterances from Time 1 to Time 2. Meanwhile, the intercept of NCIU was also significant, which means that there was a significant difference of baseline NCIU among the children. The random effect was not significant. The growth pace from Time 1 to Time 2 seemed to be the same for the children in this study.

### Lexical measures

Children demonstrated growth in their lexical skills. Time was the only significant predictor of the total number of words (TNW, Figure 1). The coefficient was 84.69 and an increase of 85 words was observed from Time 1 to Time 2. Notably, the random effect of time was also found to be significant (*p* = 0.024). This indicates that the rate of change varied significantly among different participants, with individual children showing different growth patterns.

As to the number of different words (NDW, Figure 1), both time and gender were significant predictors. The coefficient of time was 27.38. Children demonstrated an increase of 27 different words from Time 1 to Time 2. Girls produced 14 different words more than boys. On average, girls produced 115 different words (*SD* = 21.46), while boys produced 100 (*SD* = 25.09). However, it is worth noticing that there was great variability within each gender group as evidenced by the standard deviations. Even though on average girls produced more different words, the inner group variability should be considered. Similarly, the random effect of time was also found to be significant (*p* = 0.034), suggesting significant different growth patterns among different participants.

### Grammatical measures

Upon entering preschool, children demonstrated the ability to combine 3 to 4 words into phrases and sentences, albeit with some instances of missing words and grammatical errors, as expected based on the literature (Castilla-Earls & Eriks-Brophy, 2012; Simon-Cereijido & Gutiérrez-Clellen, 2007). Both time and gender predicted the mean length of utterance in words (MLUw, Figure 1). MLUw exhibited an increase of 0.57 words per utterance from Time 1 (*M* = 3.4, *SD* = 0.48) to Time 2 (*M* = 3.97, *SD* = 0.58). Thus, by Time 2, children produced utterances that included almost 4 words in length. Girls scored 0.36 points higher than boys in MLUw. Girls’ average MLUw was 3.89 (*SD* = 0.54), whereas boys’ average MLUw was 3.53 (*SD* = 0.66) at Time 1.

In terms of the proportion of utterances with verbs (PUV, Figure 1), time also emerged as a significant predictor. However, the increase in such utterances from Time 1 to Time 2 was relatively small, rising from 1% (*SD* = 0.01) to 2% (*SD* = 0.03).

As children progress in their language development, they are expected to omit fewer necessary words. The number of omitted words (NOW, Figure 1) was predicted, revealing time, again, as a significant negative predictor with a coefficient of −3.15. This indicates that, on average, children omitted 3 fewer words at Time 2 than Time 1. The average NOW at Time 1 was 6.96 (*SD* = 5.43), while at Time 2 it decreased to 3.81 (*SD* = 2.97).

Finally, the proportion of grammatical utterances (PGU, Figure 1) provided further insights into Spanish language development. Four significant predictors were identified in predicting PGU: time, gender, mothers’ enculturation and acculturation. PGU increased by 5% from Time 1 to Time 2, rising from 82% to 87%. Girls demonstrated a higher level of grammaticality, scoring 2% higher than boys. Interestingly, both mothers’ enculturation and acculturation positively influenced children’s grammaticality with the coefficient for enculturation to be 0.09 and the coefficient of acculturation to be 0.04. This means that when mothers exhibited a strong cultural orientation towards both Mexican and American cultures, children produced a greater number of grammatical utterances.

### Qualitative analysis of nonstandard grammatical productions

Two grammatical measures, namely the number of omitted words and the proportion of grammatical utterances, indicate that children exhibited a range of ungrammatical productions at both time points. Table 4 provides the means and standard deviations for specific nonstandard instances of articles, clitic pronouns, copula verbs, other verbs, and prepositions in individual samples at each time point. These nonstandard productions encompassed both omission and commission errors. Paired *t*-tests suggested that children significantly improved grammaticality at Time 2 with the production of articles (*t*(25)=3.239, *p* =.003, *d* =.63) and copula verbs (*t*(25)=2.038, *p* =.052, *d* =.40). However, no significant differences were observed in the number of errors with object clitic pronouns, other verbs, and prepositions between the two timepoints.

Table 5 presents the frequency of nonstandard productions of articles, copula verbs, and prepositions across the entire sample at each time point. At Time 1, omission errors were more prevalent in copula verbs and prepositions. At the noun phrase level, gender mismatches in articles occurred more frequently compared to omissions or number mismatches. After one year, the sample exhibited an increase in substitution errors for copula verbs and a persistent prevalence of omission errors over substitutions for prepositions. Regarding articles, gender mismatches continued to be more common than number mismatches and omissions. Examples of children’s nonstandard productions can be found in Table 6.

## Discussion

We analyzed the changes in the Spanish lexical and grammatical skills of 26 young Spanish–English DLLs over one year, starting from the beginning of their first year of preschool. We focused on a variety of lexical and grammatical measures from conversational speech samples, including 1) the number of complete and intelligible utterances (NCIU), 2) the total number of words (TNW), 3) the number of different words (NDW), 4) the mean length of utterance in words (MLUw), 5) the proportion of utterances with verbs (PUV), 6) the number of omitted words (NOW), and 7) the proportion of grammatical utterances (PGU). These outcomes allowed us to explore whether these young children maintained productive use of Spanish with a diverse lexicon and demonstrated growth in grammatical abilities. We employed multiple linear models to examine how various demographic, social, and individual variables, such as age, gender, and maternal enculturation and acculturation levels, predicted the participants’ Spanish lexical and grammatical skills over time. By utilizing these models, we could assess these variables’ influence on the outcome proficiency measures of interest.

### Changes in children’s spanish lexical and grammatical skills between 3;7 and 4;7

Unlike studies that have documented an apparent loss of Spanish lexical and grammatical skills as children enter preschool or elementary school (Castilla-Earls, Auza, et al., 2020; Hiebert & Rojas, 2021; Morgan et al., 2013), our study shows that entering the school system and receiving English-language instruction does not result in an abrupt dominant language shift or Spanish attrition, at least over one year and in a context characterized by substantial numbers of Spanish-speaking immigrants such as Los Angeles. We must acknowledge that our results may stem from our research context, and hence may not be generalizable to other Spanish–English bilingual children in the US who may not have access to Spanish through a variety of sources in their community.

In terms of language output, children became significantly more productive in Spanish at Time 2, both at the utterance and word level, regardless of age, gender, and mothers’ cultural orientation. Specifically, on average, children produced 10 more complete and intelligible utterances and employed 27 more different words within a set of 100 multi-word utterances between Time 1 and Time 2, despite one year of English-only instruction. To further explore the nature of utterance output, after completion of the analyses, we calculated the average percentage of intelligible utterances at each time point. On average, 88% of the utterances at Time 1 were deemed intelligible, while this percentage increased to 91% at Time 2, indicating some expressive growth. Additionally, we examined the occurrence of code-switching within the set of 100 utterances. Children exhibited a similar number of utterances with code-switching at both time points, with an average of 14 utterances at Time 1 and 13.4 at Time 2. These findings suggest that the observed growth in productivity is primarily associated with enhanced intelligibility, as a comparable number of utterances with code-switching were excluded from the analysis set at both time points.

It is important to acknowledge that our study had an observation period of one year, unlike previous studies that have examined language skills over a longer duration. This limited timeframe may not have been sufficient to observe attrition in language proficiency. Prior research investigating Spanish–English DLLs entering preschool has often demonstrated a deceleration in the development of Spanish skills concurrent with accelerated growth in English skills, with studies following children for more than a year documenting an initial linear growth in Spanish lexical and syntactic measures (Hammer et al., 2008, 2012; Hoff et al., 2018). Hence, it is crucial to consider the likelihood that as children progress in an English-speaking educational environment, there may be a decline in the pace of their Spanish development. Indeed, research focusing on Spanish–English-speaking children aged six and older consistently demonstrates a deceleration and attrition in their Spanish proficiency.

One crucial observation is that the Spanish productions of the children at Time 2 were still far from reaching full development. There are few published longitudinal studies of Spanish monolingual preschoolers’ spontaneous speech samples using a comparable methodology (Fernández Vázquez & Aguado Alonso, 2007). Yet, the progress in language production attested for our participants aligns with expected developmental patterns found in studies of Spanish monolingual children (Auza Benavides & Chávez Luján, 2013; Camargo-Mendoza & Garayzábal-Heinze, 2015). For instance, in a study of morphosyntactic development of two groups of 33-to-52-month-old children learning two dialectical varieties of Spanish (21 children learning Mexican Spanish and 12 learning Castilian Spanish), language samples collected during play reveal an MLUw range between 3.06 to 5.5; however, only 36% of the children had MLUw from 3;06 to 3;90, as observed in this study, while the rest of the participants produced longer utterances (Johnson, 1996). Another study of Colombian children between 10 months and 6;7 years of age examined MLUw in spontaneous speech across different age intervals (Camargo-Mendoza & Garayzábal-Heinze, 2015). The mean MLUw of the 11 children who were between ages 3;7 and 4;6 was 3.64 (*SD* = 0.36), comparable to our findings. A study of narratives produced by children from Mexico City also reports values for 24 four-year-old children (Auza Benavides & Chávez Luján, 2013). The MLUw mean was 4.75 with an *SD* of 1.80, with a range from 0.63 to 7.74 words. However, MLUw are known to be longer in narration than in play-based samples. This study also evaluated NDW (mean = 66.86, SD = 24.54, range = 16-123), which was lower than what we found in this study. Similarly, a study of spontaneous measures based on narrations of Colombian children found that for 37 three-year-old and for 39 four-year-old children, the means of MLUw were 4.76 (*SD* = 1.23) and 5.96 (*SD* = 1.11), respectively (Castilla-Earls & Eriks-Brophy, 2012). Of note, the reported ranges for the two age groups were quite large: for the age 3 group, MLUw ranged from 2.00 to 7.41, and for the age 4 group, it ranged from 4.15 to 8.08. Furthermore, in a longitudinal study of 50 Spanish young children, MLU measures collected at age 3, 3;6, and 4 showed a significant increase every 6 months. However, on average, the annual increase was smaller than one word and was considered relatively small (Fernández Vázquez & Aguado Alonso, 2007). Overall, then, our results align with those found in studies of monolingual Spanish-speaking children.

Although the children in our study produced longer utterances and more verbs compared to Time 1, the percentage of utterances with verbs remained low at both time points. Specifically, the percentage was 1% at Time 1 and only 2% at Time 2, indicating that the children primarily generated phrases or sentence fragments during their interactions with the research assistants. The low percentages of utterances with verbs suggest that the children rarely produced full sentences. Johnson (1996) also found that young Spanish-speaking children produced a reduced number of different verbs during play (e.g., 232 different verbs among 3067 total utterances), a result that resonates with our findings. It is also worth considering that the data collection context, which involved play sessions with natural interaction between the research assistant and the children, may have impacted the children’s use of complete clauses. While this context provides ecological validity and reflects real-world language use, it might not have strongly encouraged the production of full clauses.

Furthermore, it is important to note that the slopes for the total number of words and the number of different words were significant, implying that different children exhibited varying development rates. Due to the limited sample size, we cannot pinpoint specific factors contributing to this individual variation. However, it is plausible that similar to previous studies (Rojas & Iglesias, 2013), the initial levels of lexical and grammatical skills at Time 1 may have shaped the differential developmental rates observed among the children.

Our analysis of the changes in the proportion of grammatical utterances and the number of omitted words over time provides additional evidence that language productions at Time 2 were still undergoing development. Throughout the study, there was an improvement in grammaticality, characterized by a significant increase in the production of grammatical utterances (from 82% to 87% of the samples) and a decrease in the number of omitted words (from approximately 7 to 4 words per sample). Notably, the children in our study exhibited higher percentages of grammaticality in their speech samples compared to previous studies with preschoolers (Hiebert & Rojas, 2021), although differences in methodologies could contribute to some of these variations. In fact, Castilla Earls and Eriks-Brophy (2012) found that monolingual Spanish-speaking three- and four-year-olds were ungrammatical in 22% and 14% of the sample on average, respectively.

Nevertheless, it is important to highlight the persistent presence of nonstandard productions previously documented in the literature on language attrition after one year of preschool education in English. Specifically, while errors in articles and copula verbs demonstrated a significant decrease over time, errors in verbs, object clitic pronouns, and prepositions remained consistent throughout the study. Furthermore, we observed ongoing difficulties with noun phrase structures, particularly in the use of articles, where gender mismatches were more prevalent compared to number substitutions and omissions. These findings are consistent with previous research that has reported similar patterns. For example, a study by Morgan et al. (2013) with older children also noted a higher incidence of gender substitutions (8%) in articles compared to number omissions (3%).

Monolingual Spanish-speaking children typically acquire basic grammatical gender, particularly determiner/noun agreement, by age three (Anderson, 1999b; Montrul & Potowski, 2007). In Spanish, gender marking is generally reliable, as most nouns explicitly indicate their gender by canonical *-a/-o* word markers (Pérez-Leroux et al., 2023). Monolingual Spanish children demonstrate early mastery of gender use in articles, with two-year-olds achieving high accuracy rates of 97-100% in naturalistic speech (Snyder et al., 2001). However, our findings suggest that reduced exposure to Spanish leads to prolonged development in the acquisition of grammatical gender among Spanish–English-speaking children, which aligns with previous research (Anderson, 1999a, 1999b; Anderson & Márquez, 2009; Hiebert & Rojas, 2021; Martinez-Nieto & Restrepo, 2023). For instance, studies involving older bilingual children and using elicited tasks have identified delays in the production of articles in Spanish (Morgan et al., 2013), and children’s English proficiency levels have been found to predict the accuracy of Spanish articles (Castilla-Earls, Pérez-Leroux, et al., 2020). In elicitation studies, children often exhibit high performance but tend to favor masculine assignment (Martinez-Nieto & Restrepo, 2023; Pérez-Pereira, 1991).

In this longitudinal study, the participants were younger and in earlier stages of English learning compared to the bilingual participants in previous studies that have reported gender agreement mismatches. We conducted a comprehensive analysis to gain a deeper understanding of the nonstandard productions within our sample. Across both time points, our findings revealed that 58% of the gender mismatches in articles involved incorrect female assignments (using a feminine article with a masculine noun), 35% involved incorrect male assignments (using a masculine article with a feminine noun), and 7% of the responses were unscorable (e.g., the use of the neutral article “lo”). Specifically, at Time 1, 64% of the article gender mismatches corresponded to incorrect female assignments (to masculine nouns), and this percentage decreased to 50% at Time 2. Interestingly, this trend contradicts previous studies that frequently reported male assignments to female nouns, positing the male gender as the default noun gender in Spanish (Goebel-Mahrle & Shin, 2020). However, it is worth noting that not all studies, especially those analyzing naturalistic language samples, have observed this pattern (Pérez-Leroux et al., 2023). Moreover, early studies of Spanish development revealed an overgeneralization of feminine gender to masculine nouns (Hernández-Pina, 1984; Pérez-Pereira, 1991). Regarding number mismatches in articles, they occurred less frequently than gender substitutions. In 69% of the cases, children did not apply plural marking to the articles, resulting in errors of number agreement.

Regarding nonstandard substitutions for copula verbs, approximately 60% of the errors involved using singular forms instead of plural forms, while the reverse (plural for singular) was not observed. These findings are in line with the results of Spanish monolingual and bilingual development research, which has also shown that Spanish L1 learners (Radford & Ploennig-Pacheco, 1995), Spanish–Euskera bilingual children (Ezeizabarrena, 1997), and Spanish–English bilingual children (Silva-Corvalán & Montanari, 2008) acquire singular forms before plural forms and substitute plural verb forms with singular ones in the early stages of development. Tense and person substitutions were infrequent, with only one instance of each. Incorrect assignment of *ser/estar* was also rare, with only five observations (three instances of using *estar* instead of *ser* and two instances of using *ser* instead of *estar*).

#### Variables predicting children’s spanish lexical and grammatical skills

In terms of the variables predicting children’s Spanish proficiency outcomes, gender significantly predicted mean length of utterance and proportion of grammatical utterances. Specifically, girls exhibited a higher number of different words, produced significantly longer utterances, and demonstrated overall greater grammaticality in Spanish than boys after one year of English schooling. These findings align with research showing a female advantage in HL acquisition (Arriagada, 2005; Portes & Hao, 1998; Portes & Rumbaut, 2001; Rojas & Iglesias, 2013; Zentella, 1997). However, they contrast with the results of other studies that did not find this advantage in similar populations (Hammer et al., 2009; Uchikoshi, 2006). Our findings may reflect the documented pattern of girls exhibiting more advanced linguistic skills than boys, as observed in the monolingual literature (Fenson et al., 1994; Huttenlocher et al., 1991). Additionally, these results may stem from socialization and language use practices associated with traditional gender norms, which could lead to greater immersion and encouragement for girls to use Spanish at home (as speculated by Hammer et al., 2009). Regardless, our findings have practical implications, suggesting that boys are at a higher risk of experiencing a Spanish acquisition slowdown once they start preschool. Furthermore, these findings highlight the importance of attending to boys’ Spanish vocabulary development, as it is closely linked to their Spanish grammatical skills, particularly in relation to grammatical gender agreement in noun phrases (Kaltsa et al., 2019; Nicoladis & Marchak, 2011). Therefore, educators and families should nurture boys’ Spanish vocabulary growth, which in turn will foster their Spanish grammatical skills.

Interestingly, maternal cultural orientation predicted only one of the children’s Spanish proficiency outcomes: the proportion of grammatical utterances. Both mothers’ levels of enculturation and acculturation were significant predictors of the grammaticality of the Spanish utterances produced by the children between the ages of 3;7 and 4;7. The influence of maternal orientation to Latinx culture on children’s Spanish grammatical skills aligns with expectations, as more enculturated mothers tend to provide greater exposure to the HL, leading to more advanced language skills in that language for their children (Cote & Bornstein, 2014). However, it was unexpected that maternal orientation to American culture also predicted children’s grammaticality in Spanish. Typically, parental acculturation to mainstream society implies a greater focus on English acquisition and usage, resulting in children being more exposed to English than the HL (Phinney & Flores, 2002). Nevertheless, acculturation and enculturation are not the opposite ends of one culture continuum and individuals can have high levels of both promoting their children’s bilingualism. Indeed, according to Berry’s (2006) model of acculturation, integrated families, which are considered bicultural and bring together aspects of both languages, including speaking both English and Spanish, are the most successful in fostering their children’s acquisition of both the societal and HL. Farver et al. (2006), in particular, found that bicultural or integrated parents promoted home literacy experiences in both English and Spanish and had children who performed well on language assessments in both languages. Therefore, it is possible that the mothers in our study who exhibited high levels of cultural orientation to both Latinx and American culture were particularly invested in and successful at promoting bilingualism (both Spanish and English) in their children. These mothers may have possessed the necessary language skills to provide complex and varied Spanish input while also being acculturated to Anglo-American practices that value verbal communication, grammaticality, and bilingualism. However, further research is needed to gain a deeper understanding of how maternal enculturation and, especially, acculturation impact children’s development of the HL.

## Conclusion

In conclusion, our study shows that the Spanish productions of Spanish–English-speaking preschoolers became more numerous, intelligible, lexically diverse and grammatical between 3;7 and 4;7, suggesting that these children continue to develop Spanish even after entering English-only preschool. At the same time, children’s Spanish productions at four and a half were mostly limited to sentence fragments and contained errors in areas that have been shown to be difficult for heritage learners of Spanish, such as grammatical gender, verb morphology, clitic pronouns, and prepositions. Girls had an advantage over boys in HL development, as attested by the higher lexical diversity, mean length of utterance, and grammaticality of their Spanish productions. Both maternal enculturation and acculturation predicted the grammaticality of children’s utterances, suggesting that mothers with high levels of orientation to both Latinx and American culture may be the most successful at promoting Spanish in their children in the US context.

Our results have practical implications for educators, providers, and parents of Spanish–English-speaking children. First, entering preschool does not appear to halt children’s Spanish acquisition, especially in contexts with large numbers of recently-arrived Spanish-speaking immigrants. This means that Spanish-speaking families should continue to be encouraged to enroll their children in preschool, which will promote their English acquisition and success in the wider society. At the same time, parents and caregivers should be made aware of the risks of HL loss and the benefits of continuing to expose children to Spanish. Parents would particularly benefit from learning how to effectively engage children in language and literacy-related activities in Spanish, and how Spanish proficiency relates to English competence. Furthermore, families should be encouraged to consider schooling options that include both Spanish and English in order to ensure their children’s continued development and maintenance of Spanish. Boys and parents of boys should be encouraged to follow these recommendations given the gender differences in HL development. Furthermore, mothers should be provided ways to increase both their acculturation and enculturation levels so that they can develop the optimal skills to promote not only their children’s Spanish but also English proficiency.

As with all studies, our investigation has some limitations. First, the sample was of moderate size and set in Los Angeles, a context characterized by large numbers of Spanish speakers that may not be characteristic of other areas in the US. Given the heterogeneous nature of the Spanish-speaking population in the country, future studies should include larger samples of children from different Spanish–English bilingual communities, as children who have access to fewer Spanish sources may display less growth or even attrition in their Spanish skills as they enter the educational system. Second, we followed children only over one year, and we only documented developmental changes in Spanish lexical and grammatical skills that occurred within this time frame. It is possible that as children continue to be educated exclusively in English, their Spanish skills will show deceleration. Another limitation of this study is that, despite including gender as a predictor of children’s Spanish skills, the sample did not include a balanced number of boys and girls since the original study from which the data were drawn relied on snowball sampling for subject recruitment. Future studies should examine Spanish–English bilinguals’ HL development in a more systematic way, including equal numbers of boys and girls, tracking children’s language skills with both experimental and naturalistic methods over a longer period of time, and possibly documenting changes from age two, when home language should still be dominant, to age three, when exposure to English in preschool begins, to the outset of formal schooling in kindergarten. It is particularly important that future studies be longitudinal because only this methodology can show whether Spanish skills exhibit growth, attrition or even loss in this population at this age. We also only considered certain demographic, social, and individual variables that may predict Spanish growth or attrition. Future research should include more child-internal and external variables that may be related to the development of Spanish as an HL, including language exposure and use (i.e., language input and output), language proficiency at the beginning of the study, as well as broader sociolinguistic variables besides maternal cultural orientation such as maternal education and years in the US. Despite these limitations, it is hoped this study contributes to a better understanding of HL development in Spanish–English-speaking children.

## Supplementary Material

Supplementary material

## Figures and Tables

**Table 1.** Children’s demographic and language use information, and mothers’ education and cultural orientation

ID#	Gender	Age	Mother-to-Child Language	Child-to Mother Language	Siblings Language	Mother’s Education	Mother’s Mexican Orientation Score	Mother’s American Orientation Score
001	M	3;4	Spanish	Spanish	Mostly Spanish	Secondary	4.06	2.31
002	F	3;9	Spanish	Mostly Spanish	NA	Secondary	4.71	1.54
003	F	3;11	Spanish	Mostly Spanish	Spanish	Secondary	4.71	1.92
004	M	3;10	Spanish	Mostly Spanish	English	Secondary	4.47	1.69
005	M	3;9	Spanish	Spanish	Mostly Spanish	Primary	4.76	1.08
006	F	3;1	Spanish	Spanish	NA	Primary	4.47	1.77
007	M	3;6	Spanish	Spanish	Spanish	Primary	4.88	2.62
008	F	3;3	Spanish	Spanish	NA	Secondary	4.47	2.54
009	F	3;5	Spanish	Mostly Spanish	English	Secondary	4.29	2.62
010	F	3;10	Spanish	Spanish	Mostly Spanish	Secondary	4.76	1.54
011	M	3;10	Mostly Spanish	Mostly Spanish	English	Secondary	4.12	2.08
012	F	3;2	Spanish	Mostly Spanish	Spanish	Primary	5.00	3.15
013	M	4;1	Spanish	Mostly Spanish	NA	Comm College	4.53	3.08
014	F	3;8	Spanish	Spanish	Mostly Spanish	Secondary	4.24	2.15
015	F	3;8	Mostly Spanish	Mostly Spanish	NA	Secondary	3.18	4.23
016	F	3;6	Spanish	Spanish	Mostly Spanish	Primary	4.29	1.85
017	M	4;0	Spanish	Mostly Spanish	NA	Secondary	4.59	2.23
018	F	3;3	Spanish	Spanish	Mostly Spanish	Secondary	4.71	2.08
019	M	3;10	Mostly Spanish	Mostly Spanish	NA	Primary	4.76	1.08
020	M	3;4	Mostly Spanish	Spanish	Mostly Spanish	Secondary	4.59	2.38
021	F	3;3	Mostly Spanish	Mostly Spanish	Mostly English	Secondary	3.94	2.77
022	F	3;6	Mostly Spanish	Mostly Spanish	Mostly Spanish	Secondary	4.59	3.85
023	F	3;10	Spanish	Mostly Spanish	Spanish	Secondary	4.71	2.77
024	M	3;8	Spanish	Mostly Spanish	Spanish	Secondary	4.24	3.15
025	F	3;6	Spanish	Spanish	Mostly Spanish	Secondary	4.71	1.08
026	M	4;1	Spanish	Mostly Spanish	English	Primary	4.59	2.08

**Table 2.** Means and Standard Deviations of Outcome Variables from Time 1 and Time 2

Outcome variable		Mean	SD
Number of complete and intelligible utterances (NCIU)	Time 1	76.23	13.444
	Time 2	86.42	12.931
Total number of words (TNW)	Time 1	259.77	60.366
	Time 2	344.46	75.327
Number of different words (NDW)	Time 1	95.54	18.732
	Time 2	122.92	20.57
Mean length of utterance in words (MLUw)	Time 1	3.4	0.481
	Time 2	3.97	0.583
Proportion of utterances with verbs (PUV)	Time 1	0.01	0.014
	Time 2	0.02	0.026
Number of omitted words (NOW)	Time 1	6.96	5.429
	Time 2	3.81	2.967
Proportion of grammatical utterances (PGU)	Time 1	0.82	0.113
	Time 2	0.87	0.064

**Table 3.** Coefficients of Fixed Effects for all Mixed Linear Models

	NCIU	TNW	NDW	MLUw	PUV	NOW	PGU
Intercept	111.79*	187.21	48.55	1.30	−.001	11.41	.44
Time	10.19**	84.69***	27.38***	.57***	.01*	−3.15*	.06*
Gender	−3.60	−41.66	−14.17*	−.36*	.006	.81	−.05*
Mothers’ Enculturation	1.09	17.34	11.50	.13	.0006	−2.25	.09*
Mothers’ Acculturation	.84	1.59	−.31	.04	−.001	−1.59	.04*
Age at Time 1	−.94	.21	.05	.04	.0001	.21	−.002

* p<0.05.

** p<0.01.

*** p<0.001.

*Note:* Number of complete and intelligible utterances (NCIU); Total number of words (TNW); Number of different words (NDW); Mean length of utterance in words (MLUw); Proportion of utterances with verbs (PUV); Number of omitted words (NOW); Proportion of grammatical utterances (PGU).

**Table 4.** Means and Standard Deviations of Nonstandard Grammatical Productions in Each Sample at Time 1 and Time 2

	Time 1		Time 2			
	Mean	*SD*	Mean	*SD*	*p*	*d*
Articles	3.97	3.36	1.81	1.60	0.003	0.63

Object clitic pronouns	0.85	1.08	1.08	1.49	0.523	−0.13

Copula verbs	2.61	4.60	0.77	1.07	0.052	0.40

Verbs	2.73	2.84	2.30	1.72	0.121	0.31

Prepositions	2.77	2.34	2.27	1.80	0.330	0.19

**Table 5.** Frequency of Nonstandard Article, Copula Verb, and Preposition Productions in the Complete Sample at Each Time Point

	Time 1	Time 2
Articles (total)	96	41
Omissions	38	12
Gender mismatches	47	21
Number mismatches	11	8
Copula verbs (total)	68	20
Omissions	58	7
Substitutions	10	13
Prepositions (total)	67	54
Omissions	47	33
Substitutions	20	21

**Table 6.** Examples of Nonstandard Productions

Grammatical form	Nonstandard example	Standard target
Articles		
Gender mismatch	* C ***una*** conejo. * “*a (fem.) rabbit (masc.)*”	C ***un*** conejo. “*a (masc.) rabbit (masc.)*”
	* C Está en ***el*** cama. * “*(it) is on the (masc.) bed (fem.)*”	C está en ***la*** cama. “*(it) is on the (fem.) bed (fem.)*”
Number mismatch	* C sí (para) para ***la*** bebés. * “*yes, (for) for the (sing.) babies (pl.)*”	C sí (para) para ***las*** bebés. “*yes, (for) for the (pl.) babies (pl.)*”
	* C Es el día de ***la*** abuelitas. * “*It is the day of the (sing.) grandmas (pl.)*”	C Es el día de ***las*** abuelitas. “*It is the day of the (pl.) grandmas (pl.)*”
Omission	* C Y yo voy a hacer taco. * “*And I will make Ø taco*”	C Y yo voy a hacer ***un*** taco. “*And I will make a (masc.) taco*”
	* C ¿Por qué tiene largo pelo? * “*Why does (he) have long hair?*” [* “*Why is Ø hair long?*”]	C ¿Por qué tiene largo ***el*** pelo? “*Why does (he) have long the hair?” [“Why is his hair long?]*
Copula verbs		
Substitution	* C ***Es*** listo. * “*(It) is ready” [It is used to be ready]*	C ***Está*** listo. “*(It) is ready” [It is currently ready]*
	* C ¿Dónde ***están*** cuchillo? * “*Where are (pl.) knife?*”	C ¿Dónde ***está*** el cuchillo? “*Where is (sing.) the knife?*”
Omission	* C ¿Qué esto? * “*What Ø this?*”	C ¿Qué ***es*** esto? “What is this?”
	* C El niño así. * “*The boy Ø like this*”	C El niño ***es*** así. “*The boy is like this*”
Prepositions		
Substitution	* C (Una yo yo yo) yo tengo este huevo ***a*** casa. * “*(one I I I) I have this egg to home)*”	C (Una yo yo yo) yo tengo este huevo ***en*** casa. “*(one I I I) I have this egg at home*”
	* C Allá está (a la) ***a*** la sala. * “*(There (it) is (to the) to the room)*”	C Allá está (a la) ***en*** la sala. “*(There (it) is (to the) in the room)*”
Omission	* C voy lavar los trastes. * “*((I’m) going Ø wash the dishes)*”	C Voy ***a*** lavar los trastes. “*((I’m) going to wash the dishes)*”
	* C (um) Vive su casa allí. * “*((um) (he) lives Ø his home there)*”	C (um) Vive ***en*** su casa allí. “*((um) (he) lives in this home there)*”
