# Supplementary material for: Heritage language development in Spanish–English-speaking preschoolers: Influences on growth and challenges in the first year of English-only instruction
Source: J Child Lang. Author manuscript; Available in PMC 2025 Aug 2. (PMC12085098; doi:10.1017/S030500092400045X)
Supplement: Supplementary material [file NIHMS2078129-supplement-Supplementary_material.docx]

NCIU

| **Estimates of Fixed Effects^a^** | | | | | | | |
| --- | --- | --- | --- | --- | --- | --- | --- |
| Parameter | Estimate | Std. Error | df | t | Sig. | 95% Confidence Interval | |
|  |  |  |  |  |  | Lower Bound | Upper Bound |
| Intercept | 111.798907 | 44.404255 | 26.802 | 2.518 | .018 | 20.657409 | 202.940404 |
| time | 10.192308 | 2.984016 | 30.497 | 3.416 | .002 | 4.102294 | 16.282322 |
| Gender2 | -3.607876 | 4.493786 | 26.737 | -.803 | .429 | -12.832609 | 5.616857 |
| MexOrienSc | 1.088289 | 6.400193 | 26.737 | .170 | .866 | -12.049869 | 14.226447 |
| Am.OrienSc | .844182 | 3.049547 | 26.737 | .277 | .784 | -5.415853 | 7.104218 |
| AgeMosT1 | -.948247 | .678340 | 26.737 | -1.398 | .174 | -2.340728 | .444233 |
| a. Dependent Variable: UttSet. | | | | | | | |

| **Estimates of Covariance Parameters^a^** | | | | | | | |
| --- | --- | --- | --- | --- | --- | --- | --- |
| Parameter | | Estimate | Std. Error | Wald Z | Sig. | 95% Confidence Interval | |
|  |  |  |  |  |  | Lower Bound | Upper Bound |
| Repeated Measures | Variance | 87.737615 | 38.186087 | 2.298 | .022 | 37.386684 | 205.899223 |
| Intercept + time [subject = id1] | Variance | 56.037896 | 34.647755 | 1.617 | .106 | 16.679811 | 188.266266 |
| a. Dependent Variable: UttSet. | | | | | | | |

TNW

| **Estimates of Fixed Effects^a^** | | | | | | | |
| --- | --- | --- | --- | --- | --- | --- | --- |
| Parameter | Estimate | Std. Error | df | t | Sig. | 95% Confidence Interval | |
|  |  |  |  |  |  | Lower Bound | Upper Bound |
| Intercept | 187.205326 | 242.768462 | 29.652 | .771 | .447 | -308.838282 | 683.248933 |
| time | 84.692308 | 13.371636 | 33.088 | 6.334 | .000 | 57.490244 | 111.894372 |
| Gender2 | -41.657316 | 24.574508 | 29.619 | -1.695 | .101 | -91.872270 | 8.557638 |
| MexOrienSc | 17.344711 | 34.999794 | 29.619 | .496 | .624 | -54.173019 | 88.862441 |
| Am.OrienSc | 1.593551 | 16.676612 | 29.619 | .096 | .925 | -32.483033 | 35.670135 |
| AgeMosT1 | .206950 | 3.709540 | 29.619 | .056 | .956 | -7.373035 | 7.786935 |
| a. Dependent Variable: NTW.A. | | | | | | | |

| **Estimates of Covariance Parameters^a^** | | | | | | | |
| --- | --- | --- | --- | --- | --- | --- | --- |
| Parameter | | Estimate | Std. Error | Wald Z | Sig. | 95% Confidence Interval | |
|  |  |  |  |  |  | Lower Bound | Upper Bound |
| Repeated Measures | Variance | 1088.626641 | 816.280502 | 1.334 | .182 | 250.395174 | 4732.950494 |
| Intercept + time [subject = id1] | Variance | 2471.563648 | 1091.889810 | 2.264 | .024 | 1039.745734 | 5875.116066 |
| a. Dependent Variable: NTW.A. | | | | | | | |

NDW

| **Estimates of Fixed Effects^a^** | | | | | | | |
| --- | --- | --- | --- | --- | --- | --- | --- |
| Parameter | Estimate | Std. Error | df | t | Sig. | 95% Confidence Interval | |
|  |  |  |  |  |  | Lower Bound | Upper Bound |
| Intercept | 48.547359 | 67.544786 | 28.256 | .719 | .478 | -89.755377 | 186.850095 |
| time | 27.384615 | 3.843536 | 31.841 | 7.125 | .000 | 19.554054 | 35.215177 |
| Gender2 | -14.170604 | 6.837093 | 28.217 | -2.073 | .047 | -28.170899 | -.170308 |
| MexOrienSc | 11.499446 | 9.737604 | 28.217 | 1.181 | .247 | -8.440219 | 31.439111 |
| Am.OrienSc | -.309353 | 4.639749 | 28.217 | -.067 | .947 | -9.810153 | 9.191447 |
| AgeMosT1 | .051869 | 1.032064 | 28.217 | .050 | .960 | -2.061486 | 2.165224 |
| a. Dependent Variable: NDW.A. | | | | | | | |

| **Estimates of Covariance Parameters^a^** | | | | | | | |
| --- | --- | --- | --- | --- | --- | --- | --- |
| Parameter | | Estimate | Std. Error | Wald Z | Sig. | 95% Confidence Interval | |
|  |  |  |  |  |  | Lower Bound | Upper Bound |
| Repeated Measures | Variance | 101.449823 | 68.446607 | 1.482 | .138 | 27.037027 | 380.665613 |
| Intercept + time [subject = id] | Variance | 181.192434 | 85.576523 | 2.117 | .034 | 71.799379 | 457.256016 |
| a. Dependent Variable: NDW.A. | | | | | | | |

MLUw

| **Estimates of Fixed Effects^a^** | | | | | | | |
| --- | --- | --- | --- | --- | --- | --- | --- |
| Parameter | Estimate | Std. Error | df | t | Sig. | 95% Confidence Interval | |
|  |  |  |  |  |  | Lower Bound | Upper Bound |
| Intercept | 1.302313 | 1.524237 | 43.074 | .854 | .398 | -1.771450 | 4.376076 |
| time | .567692 | .145545 | 43.938 | 3.900 | .000 | .274353 | .861031 |
| Gender2 | -.361921 | .154202 | 43.131 | -2.347 | .024 | -.672873 | -.050970 |
| MexOrienSc | .130846 | .219620 | 43.131 | .596 | .554 | -.312020 | .573713 |
| Am.OrienSc | .043084 | .104644 | 43.131 | .412 | .683 | -.167931 | .254100 |
| T1AgeMos | .036382 | .023277 | 43.131 | 1.563 | .125 | -.010556 | .083321 |
| a. Dependent Variable: MLUw.A. | | | | | | | |

| **Estimates of Covariance Parameters^a^** | | | | | | | |
| --- | --- | --- | --- | --- | --- | --- | --- |
| Parameter | | Estimate | Std. Error | Wald Z | Sig. | 95% Confidence Interval | |
|  |  |  |  |  |  | Lower Bound | Upper Bound |
| Repeated Measures | Variance | .117756 | .058808 | 2.002 | .045 | .044247 | .313385 |
| Intercept + time [subject = id] | Variance | .111917 | .060739 | 1.843 | .065 | .038630 | .324234 |
| a. Dependent Variable: MLUw.A. | | | | | | | |

PUV

| **Estimates of Fixed Effects^a^** | | | | | | | |
| --- | --- | --- | --- | --- | --- | --- | --- |
| Parameter | Estimate | Std. Error | df | t | Sig. | 95% Confidence Interval | |
|  |  |  |  |  |  | Lower Bound | Upper Bound |
| Intercept | -.001360 | .067704 | 23.758 | -.020 | .984 | -.141169 | .138448 |
| time | .012269 | .005646 | 27.597 | 2.173 | .039 | .000697 | .023842 |
| Gender2 | .005990 | .006849 | 23.649 | .875 | .391 | -.008156 | .020136 |
| MexOrienSc | .000560 | .009754 | 23.649 | .057 | .955 | -.019587 | .020707 |
| Am.OrienSc | -.001268 | .004648 | 23.649 | -.273 | .787 | -.010867 | .008332 |
| AgeMosT1 | .000124 | .001034 | 23.649 | .120 | .906 | -.002011 | .002259 |
| a. Dependent Variable: PCUttWithVerbs.A. | | | | | | | |

| **Estimates of Covariance Parameters^a^** | | | | | | | |
| --- | --- | --- | --- | --- | --- | --- | --- |
| Parameter | | Estimate | Std. Error | Wald Z | Sig. | 95% Confidence Interval | |
|  |  |  |  |  |  | Lower Bound | Upper Bound |
| Repeated Measures | Variance | .000389 | .000141 | 2.755 | .006 | .000191 | .000793 |
| Intercept + time [subject = id] | Variance | 5.029201E-5 | 9.079183E-5 | .554 | .580 | 1.461606E-6 | .001730 |
| a. Dependent Variable: PCUttWithVerbs.A. | | | | | | | |

NOW

| **Estimates of Fixed Effects^a^** | | | | | | | |
| --- | --- | --- | --- | --- | --- | --- | --- |
| Parameter | Estimate | Std. Error | df | t | Sig. | 95% Confidence Interval | |
|  |  |  |  |  |  | Lower Bound | Upper Bound |
| Intercept | 11.406773 | 12.725898 | 46.000 | .896 | .375 | -14.209132 | 37.022677 |
| time | -3.153846 | 1.180893 | 46 | -2.671 | .010 | -5.530861 | -.776831 |
| Gender2 | .807930 | 1.286913 | 46 | .628 | .533 | -1.782492 | 3.398351 |
| MexOrienSc | -2.252898 | 1.832862 | 46 | -1.229 | .225 | -5.942259 | 1.436462 |
| Am.OrienSc | -1.591147 | .873317 | 46 | -1.822 | .075 | -3.349044 | .166750 |
| AgeMosT1 | .207611 | .194260 | 46 | 1.069 | .291 | -.183415 | .598638 |
| a. Dependent Variable: OmitWd.A. | | | | | | | |

| **Estimates of Covariance Parameters^a^** | | | | | | | |
| --- | --- | --- | --- | --- | --- | --- | --- |
| Parameter | | Estimate | Std. Error | Wald Z | Sig. | 95% Confidence Interval | |
|  |  |  |  |  |  | Lower Bound | Upper Bound |
| Repeated Measures | Variance | 18.128621 | 3.780079 | 4.796 | .000 | 12.046946 | 27.280515 |
| Intercept + time [subject = id] | Variance | .000000^b^ | .000000 | . | . | . | . |
| a. Dependent Variable: OmitWd.A. | | | | | | | |
| b. This covariance parameter is redundant. The test statistic and confidence interval cannot be computed. | | | | | | | |

PGU

| **Estimates of Fixed Effects^a^** | | | | | | | |
| --- | --- | --- | --- | --- | --- | --- | --- |
| Parameter | Estimate | Std. Error | df | t | Sig. | 95% Confidence Interval | |
|  |  |  |  |  |  | Lower Bound | Upper Bound |
| Intercept | .439673 | .248379 | 46.000 | 1.770 | .083 | -.060287 | .939633 |
| time | .055423 | .023048 | 46.000 | 2.405 | .020 | .009030 | .101817 |
| MexOrienSc | .089232 | .035773 | 46.000 | 2.494 | .016 | .017224 | .161239 |
| Am.OrienSc | .039925 | .017045 | 46.000 | 2.342 | .024 | .005615 | .074234 |
| T1AgeMos | -.002112 | .003791 | 46.000 | -.557 | .580 | -.009743 | .005520 |
| Gender2 | -.051542 | .025117 | 46.000 | -2.052 | .046 | -.102101 | -.000983 |
| a. Dependent Variable: Grammaticality. | | | | | | | |

| **Estimates of Covariance Parameters^a^** | | | | | | | |
| --- | --- | --- | --- | --- | --- | --- | --- |
| Parameter | | Estimate | Std. Error | Wald Z | Sig. | 95% Confidence Interval | |
|  |  |  |  |  |  | Lower Bound | Upper Bound |
| Repeated Measures | Variance | .006518 | .002259 | 2.885 | .004 | .003304 | .012858 |
| Intercept + time [subject = id] | Variance | .000275 | .001324 | .208 | .836 | 2.186718E-8 | 3.455295 |
| a. Dependent Variable: Grammaticality. | | | | | | | |
